# Work-Related Psychosocial Demands and Resources in General Practice Teams in Germany. A Team-Based Ethnography

**DOI:** 10.3390/ijerph17197114

**Published:** 2020-09-28

**Authors:** Elena Tsarouha, Christine Preiser, Birgitta Weltermann, Florian Junne, Tanja Seifried-Dübon, Felicitas Stuber, Sigrid Hartmann, Andrea Wittich, Monika A. Rieger, Esther Rind

**Affiliations:** 1 Institute of Occupational and Social Medicine and Health Services Research, Faculty of Medicine, University Hospital Tuebingen, Wilhelmstr. 27, 72074 Tuebingen, Germany; elena.tsarouha@med.uni-tuebingen.de (E.T.); christine.preiser@med.uni-tuebingen.de (C.P.); sigrid.hartmann1@gmx.de (S.H.); monika.rieger@med.uni-tuebingen.de (M.A.R.); 2 Centre for Public Health and Health Services Research, Core Facility for Health Services Research, University Hospital Tuebingen, Osianderstr. 5, 72076 Tuebingen, Germany; 3 Institute of General Practice and Family Medicine, Faculty of Medicine, University Hospital Bonn, Venusberg-Campus. 1, 53127 Bonn, Germany; birgitta.weltermann@ukbonn.de; 4 Department of Psychosomatic Medicine and Psychotherapy, Faculty of Medicine, Medical University Hospital Tuebingen, Osianderstr. 5, 72076 Tuebingen, Germany; florian.junne@med.uni-tuebingen.de (F.J.); tanja.seifried@med.uni-tuebingen.de (T.S.-D.); felicitas.stuber@med.uni-tuebingen.de (F.S.); 5 Occupational Psychologist and Psychotherapist, Tuebingen, Germany; wittich@praktische-arbeitspsychologie.de

**Keywords:** occupational stress, micro-enterprises, primary health care teams, participant observation, focus groups, interviews, grounded theory, Germany

## Abstract

General practices are established microenterprises in Germany providing a variety of preventive and therapeutic health care services and procedures in a challenging working environment. For example, general practice teams are confronted increasingly with work-related demands, which have been associated with poor psychological and physical outcomes. It is therefore important to gain a better understanding of issues related to occupational health and safety for personnel working in the primary care setting. This study aims to gain an in-depth understanding of psychosocial demands and resources in the primary care setting. We applied an ethnographic design, comprising a combination of participating observations, individual interviews with general practitioners (GPs) (*N* = 6), and focus group discussion with practice assistants and administrative staff (*N* = 19) in five general practices in Germany. A grounded theory approach was applied to analyze all data. Our results identified psychosocial demands and resources exemplified mainly along two typical tasks in GP practices: the issuing of medical prescriptions and blood sampling. Main psychosocial demands included factors related to work content and tasks, organization of work, and the working environment. For example, daily routines across all practices were characterized by a very high work intensity including disturbances, interruptions, delegation, and the division of labor between GPs and practice staff. Work-related resources comprised the staff’s influence on aspects related to work organization and social support. The triangulation of methods and data formats allowed the disclosure of interconnectedness between these factors. Although work processes in general practices are complex and required to comply with legal regulations, there are opportunities for practice owners and practice teams to establish working procedures in ways that reduce psychosocial risks and strengthen work-related resources.

## 1. Introduction

General practices in Germany provide a variety of therapeutic and preventive health care services and procedures, including medical check-ups, vaccinations, laboratory testing, and disease management programs. They are established microenterprises, and the general practitioners (GPs) as employers are required by law to provide measures ensuring decent working conditions (see Article 153 of the Lisbon Treaty, [1]). This includes, among other things, the prevention of work-related psychosocial risks comprising demands such as poor work organization or a hazardous working environment, and the promotion of work-related resources [2,3]. General practices are not only confronted with healthcare specific challenges but also with aspects similar to other smaller and larger businesses. This includes, for example, limited financial and human resources to develop strategies for the prevention or mitigation of work-related stress [4], a highly regulated working environment [5,6], as well as financial competition from other practices and new emerging business models (e.g., medical care centers), or increasing difficulties to find successors willing to pursue a career within a complex and demanding working environment [7,8,9]. This study aims at gaining an in-depth understanding of work-related psychosocial demands and resources in general practices as an example for small enterprises.

### 1.1. Work-Related Stress in the Primary Care Environment in Germany

We apply the definitions of the Job Demands-Resources model, which takes physical, psychological, social, and organizational aspects of work into consideration [10]. Job demands are those aspects of work that can cause physiological or psychological costs. Job resources are those aspects of work that can be useful to achieve goals, help to reduce demands, or promote individual development [10]. The job demand-control model [11], the job demand-control-support-model [12], the effort-reward-imbalance-model [13], and the concept of organizational justice [14] are further well-established theoretical models helping to understand the development of work-related stress. They relate potentially harmful factors (e.g., frequent interruptions and lack of leadership) and beneficial factors (e.g., good working atmosphere and appropriate salary) to health and health-related behaviors (e.g., [15,16]). Based on these models, existing European [3] and German [17] recommendations have structured work-related psychosocial factors, comprising, e.g., “work content and task” (e.g., completeness of tasks), “organization of work” (e.g., working procedures), “social relations” (e.g., social support from colleagues and managers), “working environment” (e.g., workplace equipment), and “new forms of work” (e.g., telework) [18]. These recommendations provide a useful framework to identify and evaluate work-related demands and resources, and we will structure our findings accordingly.

Mostly using quantitative research methods, previous studies have identified and analyzed various work-related psychosocial demands in the health care setting (e.g., [19,20,21]). Compared to the general population and other professions, prior research has shown that the risk of reporting work-related stress is relatively high in health care staff [22], including GPs and practice staff [23,24,25] (subsequently, the terms “practice staff” or “practice team” include practice assistants (PAs) and administrative staff to distinguish between the physician and non-physician professions). The reasons are manifold and have, for example, been related to a very high workload, increasing working hours per week or practice characteristics (e.g., high number of patients with statutory health insurance) [24]. Furthermore, a shortage of skilled workers, the economization of the health care system, and the increase of administrative tasks can affect job satisfaction of employees and practice owners [8,26]. In a cross-sectional survey from 2007, GPs in Germany expressed to be rather satisfied with their jobs, except for income, physical working conditions, and weekly hours of work [27]. A study using the scale-based data of the European Practice Assessment showed that GPs were generally satisfied with their colleagues and practice staff [28]. Prior research has shown that work-related demands and resources differ within the primary care environment; e.g., a recent study [23] reports that female GPs showed a higher risk for emotional exhaustion compared to male GPs. Furthermore, the burnout prevalence was higher in general practitioners working in group practices compared to single practices, but group practice employees were more likely to report burnout symptoms than group practice owners [8]. A qualitative study including GPs from eight European countries identified common work-related resources such as a positive occupational profile, a long-term doctor-patient relationship, or autonomy in the workplace [29]. A quantitative study from Germany showed that the opportunity to apply their skills and expertise had the strongest associations with job satisfaction for GPs [30]. This study also showed that practice staff rated their job satisfaction higher than GPs. A good working atmosphere, opportunities for career development, clearly defined responsibilities within a diverse spectrum of activities, recognition of their performance, the support among the practice team, as well as social interactions with patients have been identified as major work-related resources for practice staff in Germany [31,32]. They have, however, also reported a number of work-related demands including high workload, dealing with unpredictable incidents, or a lack of support and/or appreciation from supervisors and colleagues [32]. Like the GPs, they rate their income as rather low.

### 1.2. Aims and Objectives

To address current health system challenges at the local level. e.g., managing new infectious diseases and dealing with the increasing morbidity due to chronic diseases effectively and sustainably, it is important to gain a better understanding of issues related to occupational health and safety for personnel working in the primary care setting [15] in order to also ensure the quality of health care [21]. This study is the first work package (WP 1) of the transdisciplinary research consortium IMPROVE*job* (Supplementary Material File S1), funded by the German Federal Ministry of Education and Research (FKZ-01GL1751A, FKZ-01GL1851D). In this research network, researchers from medical, social, and economic disciplines address questions concerning work-related stress and job satisfaction in general practice teams, using the primary care setting as an example for small enterprises [33,34]. The results of this study (WP 1) will contribute to the development of subsequent work packages of the IMPROVE*job* study comprising the development and testing of a participatory intervention for the improvement of job satisfaction and the prevention of work-related stress within primary care teams (WP 2 and 3). The intervention is expected to improve job satisfaction and reduce and prevent work-related psychological distress [35,36]. Finally, the IMPROVE*job*-Consortium will evaluate options for the transfer of the results into small enterprises of other economic branches (WP 4), which may eventually promote the prevention and reduction of work-related stress in small enterprises.

Although previous research has investigated psychosocial working conditions of GPs [27,28,37,38] and PAs in Germany [21,31,32,39], this research has mostly focused on the individual experiences of GPs and PAs. A recent study about hospital staff’s job satisfaction emphasizes, that global scales alone do not grasp job satisfaction appropriately [40]. There is a lack of in-depth analyses of potential relationships between particular psychosocial work-related demands and resources affecting entire general practice teams when balancing, e.g., legal regulations, the patient collective, the physical structure of the practice rooms, leadership tasks and behavior, and individual situations of the GPs and practice staff.

For these reasons, we chose for a team-based ethnographic research design to study daily working routines in general practices in WP1. This enabled us to gain an in-depth understanding of how specific psychosocial demands and resources in general practice teams are interrelated. During our analysis, we discovered that the interconnectedness of tasks and responsibilities was a relevant factor. For this article, we chose to elaborate on this using the examples of “issuing of medical prescriptions” and “blood sampling” as two core tasks in primary care. Both examples are subject to regulations [41] and specific guidelines [42] and are therefore not only relevant nationally but also in an international context. Similar to other European countries, in Germany, there are over-the-counter and prescription-only drugs. The latter have to be acquired in pharmacies and require medical prescriptions issued by the treating physician only, including regular monitoring and medical supervision of the patient [43]. According to legal regulations, blood collection may be delegated to PAs once legal requirements are met [37,44,45]; however, the decision as to whether blood collection is necessary and which tests are required, remains the responsibility of the physician. Both examples come along with different forms of interconnectedness of procedures and responsibilities.

The following research questions (RQ) will guide this paper:

**RQ1:**  How do general practice teams organize working procedures related to the issuing of medical prescriptions and blood sampling?

**RQ2:**  What can we learn from these two examples (issuing of medical prescriptions and blood sampling) about the interrelation of specific work-related psychosocial demands and resources in general practice teams?

## 2. Materials and Methods

In this manuscript, we focus on the most important aspects of the research methods applied. Further detailed information on background, research design, and methodology have been published in the research protocol of this study [15]. The paper fulfils the COREQ-criteria for qualitative papers [46] (Supplementary Material File S2).

### 2.1. Research Design

To study everyday working life and work-related stress in the primary care environments, we applied a qualitative approach by means of a team-based ethnographic research design comprising participant observation, individual interviews with practice owners, and focus group discussions with PAs and other staff (e.g., administrative staff and trainees). We treated each practice as a case that we tried to capture holistically through different data formats and perspectives. Participant observation allowed to capture aspects of the working day in real time and point out patterns the participants themselves are not aware of or would be unlikely to disclose in an interview (e.g., interdependence between working procedures and the structural design of the working environment) [15]. The individual interviews with the GPs included particular challenges in the areas of leadership, team, and patient care. The focus group discussions captured the collective view on work contexts, working conditions, and work processes within the practice team. As established previously [47], the triangulation of methods and data formats [48] allowed us to provide a focused and detailed evaluation of psychosocial demands and resources in the primary care environment and point out causal relationships, which would not become apparent through surveys or interviews alone.

### 2.2. Setting, Recruitment, and Ethical Considerations

To represent a variety of primary care settings, we used a purposive non-random sampling frame [49]. We expected each practice to have unique characteristics, nevertheless sharing strong similarities. We selected practices that were different in their characteristics (urban/rural teams; single/group practices; and male/female practice owners). We also decided to only include practices if the entire team agreed to participate in the study.

Therefore, we included three general practices in urban and two practices in more rural areas of North Rhine-Westphalia, a densely populated federal state in Germany. The practices comprised single and group practices (up to 6 GPs) owned and managed by male and female GPs. Practice staff comprised between 5 and 29 mostly female employees including PAs, administrative staff, and trainees. For reasons of confidentiality, we did neither collect any patient-related data nor any personal information about the general practice teams. We estimated the age of the practice owners between 40 and 60+ years, whereas the practice staff mirrored the whole age range of occupational life. While most practice owners were white, a higher proportion of persons of color could be found among the Pas. The gender balance between the practice owners was almost equal, whereas most of the PAs were female.

All five practices, initially recruited via postal invitation and telephone, are part of a general practice network associated with the Institute for General Medicine, University Hospital Essen (Germany) and had signed a letter of intent to participate in this project during the funding application for this project. Therefore, all practices invited agreed to participate in this study (no drop-outs). Ethical approval for this study was obtained from the responsible Ethics Committee of the Medical Faculty, University Hospital of Tuebingen (reference number: 640/2017BO2). All procedures performed in studies involving human participants were in accordance with the ethical standards of the institutional research committee and with the 1964 Helsinki declaration and its later amendments or comparable ethical standards. Practice staff and patients were informed about the participant observation, individual declarations of consent were signed by the practice staff, and each participant had the possibility to revoke their consent at any time over the course of the study. Furthermore, each of the three researchers collecting the data (ET, SH, ER) signed a declaration of confidentiality.

### 2.3. Preparation of the Field Work, Data Collection and Management

In preparation of the fieldwork, the transdisciplinary research consortium IMPROVE*job*, including all authors, designed an observational framework based on established recommendations [18] with a specific focus on psychosocial demands potentially relevant in the primary care environment. The framework helped the observers to identify relevant context and to better understand “what was going on”. For example, we used the framework to identify and to structure factors that were likely to be observed (e.g., actual interruptions) or that would rather be discussed during the interviews and the focus group discussions (e.g., different perceptions of interruptions).

Prior to the participant observations, the observers attended a workshop—conceptualized and conducted by B.W.—to gain further insight into the particular norms and culture of the setting. Additionally, the researchers (E.T., S.H., and E.R.) conducted a two-day trial observation in different general practices to gain first impressions of the setting, its facilities, and organizational structures, which were developed and organized by B.W. The female observers with different professional backgrounds (health sciences, health care, and sociology) could thereby explore their own role in the field and identify suitable areas for the participant observation where they would attract as little attention and disruption as possible.

Data collection (E.T., S.H., and E.R.) commenced in February 2018. Each practice was visited daily and in turn by two researchers for 2–4 h during opening hours of a whole working week (Monday to Friday) [50] to cover a variety of situations and procedures over the course of one working week (e.g., at the reception desk, in the waiting area, the laboratory, or consulting room). If appropriate, the researchers took field notes on site, and observation protocols were written subsequently. The researchers also conducted semistructured interviews (Supplementary Material File S3: questioning routes) with each practice owner (*n* = 6) and five focus group discussions with members of the practice teams (*N* = 19). The interviews and focus group discussions lasted about 45–60 min. They were recorded and transcribed word-by-word according to a simplified system [51] by a professional company. Quality checks, depersonalization and pseudonymization of all data were carried out by the team conducting the fieldwork (E.T., S.H., and E.R.). MAXQDA 2018 [52] was used for data management. Subsequent to the analysis, all quotations included in this study were translated from German into English (E.T. and E.R.). As we did not apply a conversation analysis approach [53] but focused on the overall content and meaning of the data collected, we do not expect any significant loss of meaning due to the translation.

### 2.4. Data Analysis and Quality Criteria

Applying a grounded theory approach [54], data collection and data analysis were carried out alternatingly including open, axial, and selective coding. The grounded theory approach was chosen to connect the different data formats to each other and to unveil content and topics as well as procedures and practices. It is a process of continuous abstraction of the data, which is led by established quality criteria [55]. Open coding helped getting a first and creative access to the data. During axial coding, connections between codes were analyzed and a linking concept was worked out during selective coding. The analysis was conducted by the researchers carrying out the fieldwork (E.T., S.H., and E.R.) and student assistants independently and then in weekly team sessions to ensure credibility [55] by cross-checking the interpretations. An interdisciplinary team of researchers of the IMPROVE*job*-Consortium with expertise in general (B.W.), psychosomatic (T.S.-D., F.J., and F.S.), and occupational medicine (M.A.R.) as well as sociology (C.P.) was involved in various constellations at crucial points to the analysis. This helped to enrich the theoretical perspectives that guided data analysis and to increase transferability of the results [55]. We completed the alternating process of data collection and analysis when no new conceptual insights occurred during the discussion of the material from the fifths practice [56]. To assess the resonance of the findings [55] presented in this study, E.T., S.H., and E.R. conducted a workshop with three GPs and two PAs who were not previously involved in the research process [48].

## 3. Results

In our study, we analyzed how specific psychosocial demands as well as resources in general practice teams were interrelated. Subsequently, we will present how general practice teams organize working procedures related mainly to the issuing of medical prescriptions and blood sampling. The major themes go in line with the dimensions introduced by the Joint German Occupational Safety and Health Strategy (GDA) [18]. Although we aimed to present specific examples for each of the psychosocial demands and resources included, it becomes evident that many of the quotations could also be categorized into other subsections highlighting the complexity of the working procedures described.

**Figure 1:**
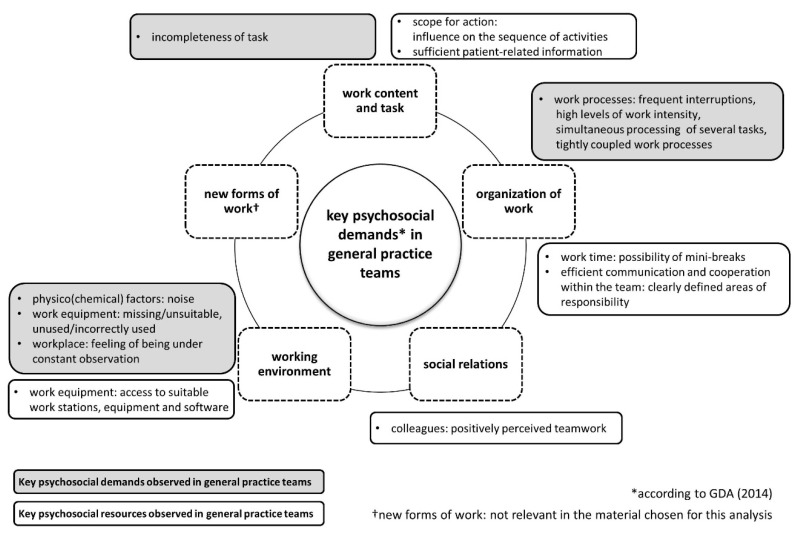
Work-related psychosocial demands, risks, and resources in general practice teams.

As summarized in Figure 1, key psychosocial demands observed were related to work content and task (incompleteness of tasks), organization of work (frequent interruptions, high levels of work intensity, simultaneous processing of several tasks, and tightly coupled work processes), and the working environment (noise, missing, unsuitable or unused/incorrectly used equipment/software, and the feeling of being under constant observation).

Key resources were related to work content and task and comprised an appropriate scope for action (influence on the sequence of activities) and sufficient patient-related information, particularly during the process of blood sampling. Furthermore, factors regarding work time (possibility of mini-breaks) and efficient communication and cooperation within the team (clearly defined areas of responsibilities particularly in the laboratory) were important resources related to work organization. In terms of social relations, positively perceived teamwork was an important resource, and a supportive working environment included access to suitable workstations, equipment, and software. Aspects related to “new forms of work” (e.g., atypical forms of employment, geographic mobility, no clear division between work and private life) [18] were not relevant in the material chosen for this analysis.

### 3.1. Work Content and Task

Responsibilities for the issuing of prescriptions as well as for blood sampling are largely determined by legal regulations and required training. In comparison to GPs, who were responsible for the entirety of the procedures, the practice staff took over primarily preparatory and executive tasks. For example, all PAs needed to be able to prepare prescriptions and take blood from patients, whereas trainees needed to be supervised and other employees (e.g., administrative staff) might support the medical staff, but were not supposed to execute tasks requiring medical training.

Two aspects became evident during the preparation of medical prescriptions: (1) the specific responsibilities of the GPs and (2) the teamwork required to complete the task. Prescriptions were issued or reissued directly as a result of the consultation with the GP, or they were reissued without an appointment. The latter occurred frequently during consultation hours when patients asked for a renewal of prescriptions by telephone, email, or directly at the registration desk. Before the prescription can be handed to the patient, the GP is required by law to check and sign the prescription [43]. Hence, the GPs can either complete the entire tasks by themselves or delegate parts of the process—the preparation and handing over of the prescription to the patient—to trained personnel [41]. Although each practice observed had slightly different ways of handling prescriptions, staff was always involved in the administrative part highlighting the division of this task through delegation and the dependency of the practice team on the GP to complete the whole procedure:

**Quotation 1, observation protocol, single practice**: *“A PA returns to her work station where a waiting patient requests a prescription. The PA immediately begins to prepare and print off the prescription. The patient is asked to wait in the waiting area until the prescription has been signed [by the GP].”*

The practice staff (and the patients) had to wait frequently for the GPs to sign prescriptions. Due to the high work intensity observed in all practices, the practice team usually turned to another task (e.g., patient registration and answering the phone), which was interrupted when the prescription was signed and had to be handed over to the patient. This example also highlights that staff were constantly meeting the needs of patients, colleagues, or superiors.

Compared to the process of issuing prescriptions, PAs carried out both administrative and medical procedures during the process of blood sampling. The blood sampling procedure usually comprised a prior consultation of the patient with the GP who ordered a specific blood test. Subsequently, the GPs could either complete the whole procedure by themselves or delegate particular tasks to a PA, including the preparation of the necessary administrative and medical equipment, the collection of the blood, and the preparation of the blood sample for the transport to an external laboratory usually undertaken by a laboratory transport service. Some tests (e.g., blood sugar) were carried out directly in the practices, whereas the comprehensive analysis of blood samples was completed by an external laboratory. Usually the PAs could execute their part of the procedure without any further consultation of the GP until the results from the laboratory arrived. Then, the GP communicated the results to the patient and was responsible for the final documentation. Compared to handling prescriptions, the PAs had a larger scope of action during the process of blood sampling including preparatory as well as executing tasks, more influence on the sequence of the work (e.g., the preparation of the equipment was usually undertaken the day before), and the probability of completing one task at a time was greater.

### 3.2. Organization of Work

As noted previously, organization of work was partly determined by legal regulations and recommendations as well as by the management preferences of the GPs and arrangements negotiated within the team. The subsequent example also highlights the importance of acknowledging the broader organizational context including an assessment of whether work content and task fit the training or abilities of the staff:

**Quotation 2, interview, single practice**: *“We used to work with ladies who have [now] retired. For them it was a nightmare to work with the computer. Or we used to have somebody […] who was almost deaf. She said: ‘I can do anything, but I can’t answer the phone.’ Ok, here I have to show consideration. But I have to have worked at another work station to appreciate the work of the others and to understand—we do that in team meetings when we have time - which part do I play in the whole system? And if I do not play my part which processes are interrupted or blocked?”*

An interruption is a temporary suspension of the current activity, which is to be continued at a later time [57]. Across all practices, we observed high levels of work intensity. Interruptions through colleagues and patients were part of many work processes, although not always consciously perceived as being particularly stressful:

**Quotation 3, focus group discussion, single practice:**

**Interviewee 1:***But I used to experience this when I was by myself at the front [desk]. […], telephone, everybody wants to pick up something, this and that. […] but if this is already stress, […], no, […].”*

**Interviewee 2**: *“Yes, because you have that [incomplete task] in the back of your mind. Hopefully, I do not forget anything […].”*

**Interviewee 3**: *“Yeah, that is stressing you out, I think so, that is stressful, isn’t it? So, hopefully I haven’t forgotten anything. Register something, prepare a bill, this and that.”*

Subsequently, a PA describes an approach to deal with interruptions:

**Quotation 4, focus group discussion, group practice**: *“Especially at the reception desk, you cannot finish a thing, you really have to put your notepad next to you and write stuff down, bullet points, because otherwise you’ll forget stuff. Of course, this shouldn’t happen, so everything should be written down immediately and if possible one thing should be completed before the next thing.”*

On the one hand, interruptions due to incoming calls or inquiries from patients were likely to be put on hold till the previous task was completed:

**Quotation 5, focus group discussion, single practice:**

**Interviewee 1:***“We try to be aware of everything. So, if somebody interrupts us at the registration desk while we are working on something else and another patient comes and says ‘I’d just like to …’, [we say:] ‘Just a moment, we’ll finish this first, because otherwise we’ll lose track and then we can [...] continue with you’. […].”*

**Interviewee 2**: *“Exactly. […]”*

**Interviewee 1**: *“[…] everybody has to queue […].”*

**Interviewee 2**: *“Everything is being taken care of one after another.”*

**Interviewee 1**: *“Exactly, exactly.”*

**Interviewee 2**: *“So that one thing can be finished.”*

**Interviewee 1**: *“Yes.”*

Although interruptions could frequently not be avoided, there was some scope for prioritization, which increased the likelihood to complete one task before the next. Prioritization was particularly relevant at the reception desk, the center of activities and interruptions during consultation hours:

**Quotation 6, observation protocol, group practice**: *“Meanwhile [on top of all of the other things happening] the phone rings, which is ignored by the trainee saying ‘this has to wait’.”*

On the other hand, interruptions from colleagues or superiors were frequently normalized:

**Quotation 7, focus group discussion, group practice:**

**Interviewer:***“[…] I got the impression there are a lot of feedback loops and inquiries [between you and your colleagues], […].”*

**Interviewee 1**: *“Yes, I don’t even notice it, it just happens, doesn’t it? We just talk it over.”*

**Quotation 8, focus group discussion, group practice:**

**Interviewer**: *“[…]. During the observations I got the impression that you are exposed to many interruptions over the course of one particular task.”*

**Interviewee 2**: *“Yes.”*

**Interviewer**: *“How do you feel about that?”*

**Interviewee 2**: *“That’s right. Yes, that’s right. Even if you always do the same thing, you can finish a lot of things, but it depends on whether [one GP] appears from a consulting room or [another GP] appears from [another consulting room], if you want to make a phone call, of course you have to interrupt this and finish it later. That’s right. That happens every day.”*

The procedure of blood sampling is an example of mostly *“do[ing] the same thing”* (Q 8). Usually, blood sampling took place during early consultation hours in a specifically equipped laboratory undertaken by one PA assigned to that task, sometimes for the entire week. As the process of blood sampling was largely prepared and executed by the Pas, we observed interruptions more frequently during the issuing of prescriptions where the practice team was more dependent on the GPs to complete the procedure. The handling of prescriptions was taken care of at various work stations throughout the practice by GPs as well as the team (e.g., registration desk, consultation room, and back office). On the one hand, GPs sometimes requested to reduce interruptions during consultation hours:

**Quotation 9, interview, group practice**: *“I’m calling a patient, […]. At that moment one of the staff dashes forward and hands a prescription to me. [...] And here I am bossy and say: ‘Dear people, please arrange work in a way that the consultation hours with the patients run smoothly and thoroughly; therefore, I do not want any heckling, for example, no telephone calls.’”*

On the other hand, we also observed that prescriptions were signed by GPs during ongoing treatments of patients interrupting the consultation process, potentially resulting in spending more time, concentration, and energy to return to the original task. For the PAs, however, this was an opportunity to complete their task quickly and hand the signed prescription to a waiting patient:

**Quotation 10, observation protocol, single practice**: *“While [the GP] is treating the patient, a PA knocks at the door and enters with a prescription for [the GP] to sign. While the [GP] is typing something at the computer, the PA is standing behind [the GP]. She has to wait a moment until the GP finishes typing and turns towards her taking the prescription to sign, handing it to the PA. The PA takes the prescription and leaves. [The GP] and [the] PA have not spoken a word to each other.”*

In some of the practices observed, there were particular areas for prescriptions to be signed (e.g., trays and shelf space), some of which were in the immediate vicinity of the consulting rooms. The GPs were able to finish an appointment or a series of consultations before signing documents such as prescriptions stored in designated trays. This enabled GPs to control their flow of work, and interruptions in the consulting room were reduced.

### 3.3. Working Environment

The previous example highlights the interrelation between work organization and working environment, the latter including, e.g., the spatial design of the waiting area, the treatment and consultation rooms and the associated work stations including any equipment. During consultation hours, the registration desk was the most exposed work station, the center of various activities including, such as short consultations between GPs, the PAs and administrative staff, the registration of patients, handling of prescriptions, arrangements of appointments, and the dealing with a multitude of phone calls:

**Quotation 11, observation protocol, group practice**: *“Today, the registration desk and the waiting area appear to be busier [than yesterday]. In the registration area, trainee 1 and 2 are making phone calls at the same time, one of the PAs is talking to patients or to a GP, the waiting room is full, and the patients seated in front of the laboratory talk to each other. From time to time, an alarm clock rings in the lab—something needs to be checked […]. Returning to the registration desk, GP A notices the list of registered patients and asks the PA: ‘Am I too fast?’ The PA replies: ‘No, that’s fine, there are more [patients] listed on the next page.’ GP A mentions to GP B that the consulting hours in the evening should not finish too late, […]. Now, the waiting room is completely packed, and three patients have to stand. Again, I notice that only a few patients say ‘hello or good day’ at the registration desk, but usually mention their concern immediately. PA 1 is always friendly and mostly replies ‘What can I do for you?’ PA 2 appears at the registration desk […], she is wearing surgical gloves, looks around and I ask whether she had time to talk about her tasks in the laboratory. She replies that she has to take care of an electrocardiogram first and leaves the registration area.”*

In some of the practices, the registration area was designed in such a way that a multitude of tasks could be carried out at the same time. In other practices, however, the design of the working environment aimed at the separation of tasks. For example, there were workplaces at the registration desk not equipped with a telephone. There were also practices where separate workplaces were set up in a back-office area to handle, e.g., administrative tasks or to take telephone calls. Similar to working in the laboratory, patient contact in these areas was limited, interruptions occurred less frequently, the parallel processing of several tasks was less likely, and the noise level was lower. At the same time, the staff working at the registration desk did not have to deal with particular administrative tasks (e.g., scanning of laboratory results) or were relieved of taking phone calls reducing the noise level during consultation hours considerably. Overall, noise levels at the registration desk were, however, not mentioned by practice staff without direct inquiry from the observers.

Staff members reported the feeling of being under constant observation at the registration desk. Working at the registration desk, all personnel was continuously exposed to inquiries from patients, colleagues, or superiors, and micro-breaks were important to relax the body and the mind:

**Quotation 12, observation protocol, group practice**: *“[The employee mentions to the observer] that she is “on display” at her work station. The patients can watch her continuously and she has to be on the spot all of the time. Therefore, she occasionally has to leave [her work station] for a short time to see something else and take a breath.”*

Working in the laboratory, the privacy of the PAs was more protected because the perceived social control through third parties (e.g., patients or superiors) did occur less frequently or not at all as the respective PA was responsible for only one patient at a time.

The working environment also included the availability and functioning of technical equipment at different work stations, such as computers, printers, and work specific software (e.g., laboratory management software). For example, the advantage of investing in several printers at different work stations facilitated smooth working processes, particularly evident in the laboratory environment. All but one of the laboratories visited were equipped with a computer, whereas a printer was available in only one lab. This printer, however, could not be used to print the required forms used in the laboratory:

**Quotation 13, observation protocol, group practice**: *“The PA tells [the observer] that she has to go [from the laboratory] to the front desk because she needs to use another printer. [The observer] follows the PA to the registration. Here, the PA puts [the required] form into an old printing device. […]. Subsequently, [the observer] follows the PA back to the lab. […]. The PA explains that patient data had to be printed on a particular form, and the printer in the lab can’t do that.”*

Furthermore, appropriate software facilitated laboratory processes including information on particular blood tests or medical prescriptions when staff could access patient data directly in the laboratory. For example, we observed that PAs checked current medication plans directly at the laboratory computer before choosing appropriate equipment to take blood from patients taking drugs affecting clotting time. This was possible if electronic patient records were available, which we observed only in some of the practices.

The laboratories were usually equipped with all utensils necessary to take blood samples (e.g., surgical gloves, antiseptics, used needle containers, or disinfectants); nevertheless, we observed that hygiene regulations were implemented to varying degrees of consistency in several practices:

**Quotation 14, observation protocol, group practice**: *“[The PA] goes to the sink, takes some disinfectant into her right hand to rub a little on her hands. Shortly afterwards, she takes paper towels and wipes her hands again. [The observer] wonders whether this was the entire procedure of hand disinfection. In any case, this did not comply with the hand hygiene protocol poster displayed at the wall […]. Furthermore, neither did she use surgical gloves during the blood sampling nor has she disinfected or washed her hands between different patients.”*

As our project did not intend to evaluate workplace hygiene, the implementation of hygiene regulations was not discussed further in any of the interviews or focus group discussions. During one observation only, a single staff member mentioned that hand hygiene gave the patient a sense of security, whereas self-protection was not mentioned at all. As we wanted the staff to discuss factors related to occupational stress as openly as possible, we aimed to avoid any criticism related to hygiene regulations.

Spatial design and missing or faulty equipment resulted in additional work for staff, particularly evident in the laboratories and at the registration desk. The availability of additional printers and appropriate software in the laboratory would facilitate work processes and avoid additional noise or work at the registration desk; from an economical point of view, however, providing additional equipment also created additional costs.

### 3.4. Social Relations

Although some of the GPs emphasized their special and long-term relationships with patients as important social relations, the PAs participating in the focus group discussions highlighted particularly the value of mutual support and team work that we observed across all practice teams:

**Quotation 15, focus group discussion, group practice:**

**Interviewee 1:***“Well, there are really days when you see, for example, that [there is a lot of work to do] in the lab. […]. The person who has [less to do] supports the person in the lab. […]. Yes, there are really situations like that. Anyhow […], you just go and help.”*

**Interviewee 2**: *“We always support each other, no matter what.”*

Across all practices observed, there were several work stations at the registration desk where PAs could work simultaneously, facilitating mutual support, but also increasing the likelihood of interrupting each other. In the laboratory, work stations were more isolated, which sometimes complicated direct communication and support. We observed, however, that PAs took particular effort and care to support each other during busy laboratory hours, which also included communication across different work stations and rooms and additional support from staff designated to different work stations than the laboratory:

**Quotation 16, Observation protocol, group practice**: *“[After trying twice] the PA stops the blood sampling and informs the patient that she will get a colleague. The patient says that this is not necessary. The PA insists and says that she only tries twice [to puncture a vein]. […]. She leaves the laboratory and after a short time a colleague appears, who […] grabs a pair of surgical gloves from one of the two boxes, and sits down at the table [to continue with the procedure].”*

Over the course of the field work, all practice teams highlighted the interdependency of each team member to deal with the work intensity and ensure the quality of patient care. It was also discussed that insufficient team work could result in a serious burden for both individual team members as well as for the entire staff.

## 4. Discussion

### 4.1. Comparison between Working Procedures (RQ 1)

Using the examples of issuing prescriptions and blood sampling, we analyzed how general practice teams organized working procedures (RQ1). The major themes of our findings are in line with the well-established GDA-recommendations defining key psychosocial demands and resources [18].

All professional groups were involved in the issuing of prescriptions and blood sampling. The practice staff usually prepared the prescriptions for the GPs to check and sign before handing the prescription to the patient. This division of labor resulted in direct interdependency of the professional groups involved. In comparison, the GPs’ delegation of blood sampling to PAs resulted in a more indirect interdependency of the professional groups involved. In terms of work organization, the issuing of prescriptions was organized in a manner that it became more prone to interruptions than blood sampling. With regards to work content and tasks, blood sampling could be completed easily as a task, while the issuing of prescription was interrupted several times before completion. Depending on the spatial design and the use of the spatial design, the issuing of prescription was either fulfilled in a noisy, busy, and exposed working environment or in a calmer and less exposed working environment, while blood sampling was always executed in calmer and less exposed working environments. Strong social relations within the teams were reported by the participants with regards to both tasks, which feeds into existing research [12,58,59].

### 4.2. Cumulative and Compensatory Interrelation of Psychosocial Demands and Resources (RQ 2)

The working conditions described for general practice teams highlight the interrelation between work-related demands and resources (e.g., balance/imbalance between work-related demands, work performance, scope for action, social support, and perceived appreciation from colleagues and patients) (RQ2). Daily routines across all practices were characterized by a very high work intensity frequently concurring with disturbances, interruptions, delegation, and the division of labor. In line with previous research [17], our results demonstrate that different psychosocial demands and resources do no occur in isolation, but are closely interconnected. Working at the reception desk is an example for the cumulative and compensatory effects of different psychosocial demands and resources. In comparison to single work stations, the reception desk is intensified with more intense working environment as several tasks and people cumulate in a rather small space that becomes bustling and buzzing. Here, all of the previously reported perceived potential demands come together including noise, frequent interruptions, the parallel processing of several tasks, and working under observation of third parties [18,60]. In our study, noise was hardly addressed by the participants, but observed and addressed by the researchers. All other factors were also addressed by the participants themselves. Especially noise and interruptions have not necessarily been reported as stressful by the participants. We interpret this as a form of normalization of specific demands and link it to the compensatory effect of working together as a team, which has already shown to be a well-established work-related resource that can mitigate factors associated with an intense working environment [12,61]. Spatial proximity can facilitate mutual support and speed up work processes. For practice staff, the compensatory effect of team work might be particularly noticeable at the reception desk as the intense working environment also intensifies interdependencies, which might be experienced as strong and strengthening mutual support in a well-functioning team. This might also bring deeper understanding to the uniqueness of the doctor–patient relationship, comprising long-term rich relationships with patients as well as mutual trust and respect, previously described as an important resource for GPs in Europe [29]. Our findings indicate that while practice staff might find frequent mutual support among team colleagues, for GPs, the support of the practice staff might be noticeable more indirectly and subtly in the form of securing smooth processes. Thus, our data support that, among other reasons, the doctor–patient relationship becomes an important resource for the GPs.

Furthermore, our study highlights that interruptions are a consequence, but also means of dealing with interdependencies in procedures that are organized with strong interconnectedness between GPs and practice staff. For example, GPs were interrupted by practice staff during ongoing consultations or other tasks to check and sign prescriptions for patients waiting outside the consultation rooms. For the GPs, the interruption was a potential demand, including loss of time and additional effort required to finish their primary task [17,62]. For the PAs, however, the interruption of the GP was a resource as it enabled a broader scope of action. It helped to finish a task quickly (dealing with a prescription and waiting patients) and decreased the likelihood of taking up another task while waiting for the GP’s signature. This means, the very same interruption can be a demand and a resource, depending on the situational context. Interruptions themselves were not seen and treated as avoidable (e.g., through organizing less interconnected procedures), but as demands and resources that needed to be balanced. Interestingly, none of the practices decided to issue repeat prescriptions after consultation hours, which could reduce the frequency of interruptions for both the GPs and the practice staff, probably mitigating the negative effects associated with interruptions [62].

The interrelation of demands and resources speaks against monocausal solutions. This becomes especially apparent in the spatial design of the work environment. We observed that in some practices, the working environment was designed in a way that allowed for areas and periods during which the signing of prescriptions could be undertaken by the GP without being disturbed during consultations. This does not necessarily turn spatial design into a resource though, if organization of work is not adjusted, GPs still have to interrupt their own tasks to sign the prescription or practice staff have to wait for the GP’s signature. In addition, in terms of blood sampling, all the necessary equipment was usually provided in the laboratory areas, but sometimes not handled in the correct way or not used at all. Our observation is consistent with prior research showing that only 20% of the medical staff in hospitals applied correct hand hygiene procedures [63]. The design of the workplace thus does not automatically lead to correct implementation of working procedures. Other studies have shown that role models as well as individual knowledge, beliefs, and physical reactions to hand hygiene were important factors [63].

### 4.3. Strengths and Limitations

Other than existing cross-sectional surveys (e.g., [21,38]) and interview studies (e.g., [29,32]), our team-based ethnography allowed to zoom into the micro-mechanisms of daily work in general practices and the related psychosocial demands and resources. The study benefited significantly from the cooperation within the transdisciplinary research collaboration IMPROVE*job* [34]. For example, the development of the observational framework included the GPs and PAs of the research support group, the members of the scientific advisory board, and the interdisciplinary research group of the IMPROVE*job*-Consortium. The combination of academic and practical expertise contributed to the development of a comprehensive observational framework relevant in the primary care setting (see [64] for comprehensive discussion of transdisciplinarity in health research). Furthermore, it facilitated the recruitment process (as we had access to a local GP network) and mitigated challenges of the ethnographic approach (e.g., disruption of daily routines through the researchers) through comprehensive familiarization with the setting ahead of the participant observations. Credibility, transferability, and resonance as quality measures [55] were achieved by the interdisciplinary team during the several stages of analysis and confirmed by an independent research support group [48].

The applied team-based ethnographic design is a methodological strength of this study, as it allowed us to gain an in-depth understanding of the working routines of entire practice teams. The various methods applied allowed us to generate data about work characteristics, (a) which were observed, (b) which were actively addressed in the interviews by the research participants, and (c) which were only addressed upon a narrative request during the interviews. Thus, applying only interviews or even only a standardized questionnaire to assess psychological demands and resources would have resulted in a less complex understanding of the interrelation between the factors described in this study. Most aspects of work happen every day while others are linked to specific days of the week. Consequently, we decided to observe each practice for one working week to grasp the full cycle of weekly routines. We cannot judge whether the weeks were particularly busy or calm due to seasonal reasons (e.g., flu or holiday season), but we surely did observe a high workload across all practices.

All practices visited were part of a general practitioner network comprising practices that are involved in teaching and training of medical students. This may have resulted in a sample including participants being particularly open and reflective concerning our study approach. Over the course of the fieldwork, it became evident that especially practice staff were used to being observed at work. Our participant observation fit into this daily routine, but was surely more intimate and more intensified than the usual observations through patients as we were also allowed access to sections of the practice where patients were not allowed. Further, we were allowed to ask questions (e.g., about work organization) that patients would not be allowed to ask. We understand the issue of social desirability more as participants’ working to rule or by the book, which is one potential course of action, among several, that people have. We assume that full working to rule would not have been upheld by the participants for a whole week if their usual behavior would differ too much from it. The participants were simply too busy to constantly pay attention to the observers. The incidents in our data in which participants did not follow protocol or regulations (e.g., hand hygiene) show that they were either not able or not intending to hide critical aspects of their routines. Furthermore, the focus groups and interviews were conducted after the observations when the participants were already familiar with the researchers and mutual trust was established.

It should also be noted that the presentation of our results includes intended and unintended “blanks” [65]. For example, the different professional backgrounds of the observers (sociology, health sciences, and health care) have contributed to a comprehensive view on reoccurring themes (e.g., interruptions or hygiene standards). Nonetheless, unintended “blanks” have occurred over the course of the observation; e.g., we did record the technical equipment or the organization of particular working procedures to varying degrees because the researchers had different priorities depending on the actual situation observed or discussed during the fieldwork. Intentional “blanks” arose where, e.g., the researchers observed issues related to the implementation of hygiene regulations but did not further explore motives for particular practices to avoid any sense of occupational hazard control, which was not the aim of our research. For reasons of patient confidentiality, the researchers also predominantly observed situations outside the treatment rooms; hence, the results focus rather on the perspectives of the practice staff and to a lesser degree on those of the GPs.

Finally, we chose to report work-related psychosocial demands and resources exemplified mainly along the preparation of medical prescriptions and the procedure of blood sampling. This means we did not capture all of the psychosocial demands and risks summarized in the observational framework based on the recommendations for implementing a comprehensive psychosocial risk assessment [18]. We therefore also did not capture all of the psychosocial risks or resources relevant in the primary care setting in this paper. For example, we did not discuss appointment scheduling which is, however, a well-known organizational task that has a significant impact on the management of consultation hours [66]. Yet, many of the resources and work design measures described here have general positive effects on reducing psychosocial demands in primary care practice teams.

## 5. Conclusions

The psychosocial risks reported in this study including high work intensity, frequent interruptions, simultaneous processing of several tasks, or tightly coupled work processes have also been reported relevant in small and medium-sized enterprises of other economic branches [3]. Prior research has also highlighted the importance of positive social relationships in small enterprises where many workers describe their working environment as a place where they were “treated as a person” or “where the boss ‘cares’” and “where people ‘look out for one another’” [67]. We emphasize that the mutual social support we observed across all practices mitigates work-related demands to a certain degree, but should not be taken for granted as a resource for coping with psychosocial demands. Our team-based ethnography in general practices has contributed to highlight that each practice is its own microcosm in which legal regulations, spatial features, patient collective, individual backgrounds of the employer and the employees, and organization of working procedures have to be balanced on a daily basis. Monocausal measures could cause more damage than good or at best shift psychosocial demands without solving them. Implementing the mandatory psychosocial risk assessment [18] in general practices as well as in all smaller and medium-sized enterprises should therefore not be seen as another administrative burden, but as an opportunity to find adequate and tailored ways to deal with the uniqueness of each enterprise and its work-related psychosocial demands and resources.

## Supplementary Materials

The following are available online at https://www.mdpi.com/1660-4601/17/19/7114/s1, File S1: Transdisciplinary research consortium IMPROVE*job*, File S2: COREQ-checklist, File S3: Questioning routes.

## Please Note

An earlier version of this manuscript with the title “We always support each other—no matter what”: a qualitative analysis of work-related psychosocial demands, stressors and resources in general practice teams was uploaded on the preprint platform Research Square (DOI:10.21203/rs.3.rs-21775/v1; DOI:10.21203/rs.3.rs-21775/v2). The here published article is the revised and finalized version of the preprint.
