# Supplementary material for: Work-Related Psychosocial Demands and Resources in General Practice Teams in Germany. A Team-Based Ethnography
Source: Int J Environ Res Public Health. 2020 Sep 28;17(19):7114. doi: 10.3390/ijerph17197114 (PMC7579545; doi:10.3390/ijerph17197114)
Supplement: Supplementary file 1 [file ijerph-17-07114-s001.zip › ijerph-908701-Supplementary/S1 - IMPROVEjob-Consortium.pdf]

Supplementary file 1: IMPROVE*job*-Consortium

Current institutions and members:

Institute of Occupational and Social Medicine and Health Services Research, University Hospital Tuebingen, Germany: MA Rieger, E Rind, A Siegel, A Wagner, E Tsarouha

Department of Psychosomatic Medicine and Psychotherapy, Medical University Hospital Tuebingen, Germany: F Junne, T Seifried-Dübon, F Stuber, A Herrmann-Werner, S Zipfel

Institute of General Practice and Family Medicine, University Hospital Bonn, Germany: B Weltermann, S Kasten, K Linden, L Degen

Operations Research, Ruhr-University Bochum, Germany: B Werners, M Grot

Institute for Medical Informatics, Biometry and Epidemiology & Center for Clinical Studies, University of Duisburg-Essen, Germany: K-H Jöckel, C Pieper, V Schröder, J-M Bois, A-L Eilerts, M Brinkmann

Former institutions and members:

Institute for General Medicine, University Hospital Essen, Germany: C Kersting

Institute of Occupational and Social Medicine and Health Services Research, University Hospital Tuebingen, Germany: S Hartmann (née Emerich), S Burgess, M Hippler

Institute of General Practice and Family Medicine, University Hospital Bonn, Germany: A Dreher

Institute for Medical Informatics, Biometry and Epidemiology & Centre for Clinical Trials, University of Duisburg-Essen, Germany: C Ose

Operations Research, Ruhr-University Bochum, Germany: L Imhoff (née Koppka), J Block
