# Supplementary material for: Work-Related Psychosocial Demands and Resources in General Practice Teams in Germany. A Team-Based Ethnography
Source: Int J Environ Res Public Health. 2020 Sep 28;17(19):7114. doi: 10.3390/ijerph17197114 (PMC7579545; doi:10.3390/ijerph17197114)
Supplement: Supplementary file 1 [file ijerph-17-07114-s001.zip › ijerph-908701-Supplementary/S3 - Questioning routes.pdf]

Supplementary file 3: Semi-structured questioning route - single interview

I. Introduction and information
**Background:**<br>[**_Mandatory_**: Thank you for your time and your cooperation. My name is [NAME] and I work at the XXX – Anonymous for review. We are cooperating with the Institute Anonymous which you support because your practice is part of their general practice teaching network.<br>We are interested in work-related stress. We would like to learn something about your day-to-day work processes and how you experience work-related stress and strain. This interview will focus on your personal work experience and perspectives. There are no right or wrong answers. Everything that is relevant to your working life is of interest to us.<br>**Tape recording and data protection:**<br>You have already been informed that our conversation will be recorded, so that we will be able to transcribe and analyze the conversation afterwards. All data will be pseudonymized; that means we never use your name or address, but a code so nobody can identify you, your practice, patients, or members of your practice team. You have read the data protection declaration and have given your consent. Do you still agree with the recording and the use of your data? You are allowed to stop the interview at any time and your interview data will be deleted at your request.<br><br>**Do you have any further questions?**<br><br>**If not, I would like to start with the interview and the recording.**
**II. Topic 1: Work organization**
**Introductory questions**
❖ *As a practice owner, you have different tasks and responsibilities. You are the owner and manager of a company, you are a doctor and you are a member of the practice team. Where do you see particular challenges?**
❖ *What is important to you regarding the organization of work in your practice?*<br>o structural workplace design**<br>o team atmosphere<br>o organization of processes
**Questions about typical work processes**
❖ *How is the cooperation with your colleagues organized (e.g. assignment of patients)?****
❖ *What is important for you when you cooperate with other doctors in the practice?****
❖ *Which aspects are important to you as a team leader?*
❖ *How do you organize sick leave?*
❖ *How do you organize holidays??*
**Questions about interaction**
❖ *What is important to you when you communicate with your team?*<br>o Communication with other doctors?***<br>o Communication with physician assistants or other staff?
❖ *As a practice team, what is important to you when you communicate with patients?*
**Questions on mutual support**
❖ *How do you perceive the support of your team?*
❖ *How do you deal with suggestions from your employees on how to organize particular work*

processes?
❖ *How do you deal with personal/professional matters of your employees?*<br>o Family: work-life balance, pregnancy<br>o Professional: integration of trainees, retirement of staff
**Questions about work intensity**
❖ *How do you experience the intensity of work, e.g. with regard to*<br>o *treatment of patients?*<br>o *multitasking?*
**III. Topic 2: Work content and task**
**Questions about the distribution of work**
❖ *How do you decide who takes over which tasks in the team?*
o Which tasks can only be overtaken by particular staff?<br>o Which task(s) is each team member required to be able to do?<br>o Where are overlapping areas of responsibility (so that certain tasks cannot be explicitly assigned to one employee)?
**Questions about emotional demands**
❖ *Which work-related matters do you take home or deal with after work?*
**IV. Topic 3: new forms of work**
**Questions on the conditions of employment**
❖ *What do you consider to be particularly important thinking about conditions of employment?*
o What is your position, for example, on (non-)fixed-term contracts?
o What is your opinion on different work time models?
o What opportunities for professional development are available to you and your practice team?
o Are you available (eg, telephone, e-mail, WhatsApp) after work?
**V. Conclusion of the interview**
❖ *Is there anything you would like to add or discuss further?*
**Thank you very much for your participation in this interview!**

Notes:
**Questions in italics are open questions;*
**bullet points to stimulate conversation
(not always asked depending on development of the interview);
***Questions only asked in group practices.

Semi-structured questioning route - group discussions

I. Introduction and information
**Background:**<br>[***Mandatory**: Thank you for your time and your cooperation. My name is [NAME] and I work at the XXX. We are cooperating with the Institute XXX which you support because your practice is part of their general practice teaching network.*<br>We are interested in work-related stress. We would like to learn something about your day-to-day work processes and how you experience work-related stress and strain. I will sometimes ask questions, but this discussion will focus on your personal work experience and perspectives you are welcome to share and to discuss with each other. There are no right or wrong answers. Everything that is relevant to your working life is of interest to us.<br>**Tape recording and data protection:**<br>You have already been informed that our conversation will be recorded, so that we will be able to transcribe and analyze the conversation afterwards. All data will be pseudonymized; that means we never use your name or address, but a code so nobody can identify you, patients, or other members of the practice team. You have read the data protection declaration and have given your consent. Do you still agree with the recording and the use of your data? You are allowed to stop the interview at any time and your interview data will be deleted at your request.<br>**Do you have any further questions?**<br>**If not, I would like to start the focus group and the recording.**
**II. Topic 1: Work organization**
**Introductory questions - picture cards ***
*Which picture card represents your role within the team? Why did you choose this card?***
**Questions about typical work processes**
❖ *Please describe your typical working day – what do you do?*
o particular challenges***
o interruptions by patients or colleagues
**Questions about interaction**
❖ *How do you communicate within the team? What is important to you?*<br>o physician assistants or other staff with each other<br>o with superiors
❖ *As a practice team, what do you consider important when communicating with patients?*
**Questions on mutual support**
❖ *How do you experience team work and support within the team?*
❖ *How does your boss support you?*
❖ *Do you have ideas and suggestions on how to improve work processes? How do you discuss suggestions and new ideas within the team and with your boss?*
❖ *How do you deal with personal/professional matters within the team?*<br>o Family: work-life balance, pregnancy<br>o Professional: integration of trainees, retirement of staff<br>❖ *How does your boss deal with personal/professional matters of you?*

Questions about work intensity
❖ *How do you experience your working day, e.g. with regard to*<br>o *time pressure?*<br>o *multitasking?*
**III. Topic 2: Work content and task**
**Questions about the distribution of work**
❖ *How do you decide who takes over which tasks in the team?*<br>o Which tasks can only be overtaken by particular staff?<br>o Which task(s) is each team member required to be able to do?<br>o Where are overlapping areas of responsibility (so that certain tasks cannot be explicitly assigned to a particular person)?
❖ *Are there particular work processes requiring more than one person to be completed?*
**Questions about emotional demands**
❖ *Which work-related matters do you take home or deal with after work?*
❖
**IV. Topic 3: new forms of work**
**Questions on conditions of employment**
❖ *What do you particularly like about the conditions of your employment?*
o type of contract
o opportunities for professional development
❖ *Are you available (eg, telephone, e-mail, WhatsApp) after work?*
**V. Conclusion of the focus group**
❖ *Is there anything you would like to add or discuss further?*
**Thank you very much for your participation in this interview!**

Notes:
*Prior to the first focus group discussion, eleven picture cards [No. 1, 9, 14, 20, 31, 57, 60, 62, 63, 71, 72] (Weidenmann and Weidenmann, 2013) were selected randomly by two researchers. These cards were presented to the participants at the beginning of each focus group discussion. To start the discussion, the participants were asked to choose a motive representative for their role in the team.
***Questions in italics are open questions;*
*** bullet points to stimulate conversation (not always asked depending on development of the focus group);

Reference

Weidenmann, S., Weidenmann, B., 2013. 75 Bildkarten für Trainings, Workshops und Teams *[75 Picture Cards for Trainings, Workshops and Teams],* Beltz Verlag Weinheim: Basel.
